# Nutritional quality of indigenous legume browse in southern Ethiopia: farmers’ preference and correlation of local valuation of feed value with scientific indicators

**DOI:** 10.3389/fvets.2023.1198212

**Published:** 2023-08-21

**Authors:** Getachew Abraham, Yisehak Kechero, Dereje Andualem

**Affiliations:** ^1^Department of Animal Science, College of Agricultural Sciences, Arba-Minch University, Arba Minch, Ethiopia; ^2^Department of Animal Science, College of Agriculture and Natural Resources, Dilla University, Dilla, Ethiopia

**Keywords:** agroecosystems, farmers’ evaluation, feed value, indigenous knowledge, nutritive value

## Abstract

**Introduction:**

Developing a technology for fodder trees and shrubs tailored to farmers’ preferences is best done with their input, perceptions, and interests in mind.

**Objective:**

The research aimed to determine farmer preferences for indigenous legumes, fodder trees, and shrubs (ILFTS) and to examine the relationship between feed valuation and scientific parameters.

**Methods:**

A focus group discussion (FGD) was conducted with 10 farmers in each agroecological zone to determine the benchmarks for the preference ratings. The respondent farmers used the preference score sheet to rate all ILFTS on an individual basis. Twenty farmers with extensive experience in ILFTS took part in the preference score rating of each plant species in each agroecosystems. Dry matter (DM), organic matter (OM), ash, crude protein (CP), neutral detergent fiber (NDF), acid detergent fiber (ADF), acid detergent lignin (ADL), metabolizable energy (ME) and condensed tannin (CT) content of the samples were determined. The standard two-stage *in vitro* Tilley and Terry method was used to measure the *in vitro* dry matter digestibility (IVDMD) and *in vitro* organic matter digestibility (IVOMD) of samples. Digestible organic matter in dry matter (DOMD) and ME values were estimated using standard models. Analysis of variance (ANOVA) was used to analyze the variation among the species in agroecosystems. Tukey HSD tests were used for mean separation.

**Results and discussions:**

Farmers evaluated the ILFTS using a variety of parameters, according to the study (feed value, growth rate, biomass output, compatibility, and multifunctionality). The farmers’ ILFTS preference score on the evaluation criteria differed considerably (*p*<0.05) with species in agroecosystems. The CP, ash, and ME values of ILFTS in the study were moderate to high although exhibited a wide variation among the species in agroecosystems. The CP content was above the minimum requirement (8%) to support the normal function of rumen microorganisms. Moreover, CP content exhibited a positive significant correlation with IVDMD, IVOMD, and DOMD, unlike CT and ADL which exhibited a negative significant correlation. Conversely, the DM, OM, CP, IVDMD, IVOMD, DOMD, and ME were shown a positive significant correlation with farmers’ feed value preference score, unlike the ADL and CT which exhibited a negative significant correlation.

**Conclusions:**

Farmers’ indigenous knowledge of feed value is therefore relevant for judging the nutritive value of the ILFTS and could complement the scientific indicators.

## Introduction

Fodder trees, and shrubs have been an essential source of forage for ruminants to complement the critical dry period feed deficit in the tropics (1). In addition to flourishing with a deep root system capable of absorbing water far from the surface, they produce considerable biomass of leaves, twigs, fruits, and pods which can bridge the feed supply gap commonly observed during dry periods (2, 3). Fodder trees and shrubs have high nutrient content and digestibility, although this varies by species and season (4, 5). In particular, the crude protein (CP) content of fodder trees is above the minimum requirement for the normal microbial function of the rumen, so it is usually recommended to supplement poor-quality fiber-based diets (6, 7). Feeding ruminants’ legumes, fodder trees, and shrubs improve the intake and digestibility of low-fiber-based diets by increasing the activity of rumen microorganisms via improving the nitrogen supply which is necessary for their proliferation (8). However, deterring mechanisms related to phenolic compounds, especially high condensed tannin (CT) content, which reduce feed intake, nutrient digestibility, and nitrogen retention, limit their potential as feed resources for herbivores (9). Condensed tannin in low to medium concentrations (below 5 g/kg dry matter) has been found to benefit ruminant production by improving rumen bypass protein and carbohydrates, preventing bloat and helminthiasis, and reducing greenhouse gas emissions (9).

Indigenous fodder trees and shrubs are adaptable to the local environment due to their pest resistance and drought tolerance. Furthermore, they are preferred to exotic browse trees due to their palatability, high nutrient value, biomass yield, readily available planting materials, and local community appreciation (10). Currently, indigenous fodder trees and shrubs have been receiving research attention in Ethiopia and other tropical countries. However, most studies ignore the farmers’ knowledge and rely on on-station agronomic and feeding trials to compare biomass yield and nutritive value of a specific species with various management practices (11, 12). This approach, however, has an impact on the spread of emerging technologies involving trees and shrubs as forage plants. The uptake of technology is determined by the farmers’ knowledge, perceptions, and attitudes, according to Meijer et al. (13). Farmers could have an immense contribution to research since their knowledge and preferences are crucial as potential users of the upcoming technologies (14). Farmers’ perceptions of trees are based on their felt needs, prior experiences, and expectations, which may or may not correspond to scientific reality (13). Boogaard et al. (15) indicated that farmers valued fodder trees based on their knowledge, experience, values, and interests.

Many countries around the world, particularly in tropical and semi-humid regions, have not dealt with studies on farmers’ preferences, nutritional quality, and their correlation with fodder trees and shrubs. However, involving farmers’ knowledge, perceptions, and interests in appraising fodder trees and shrubs and analyzing their correlation with scientifically proven methods could save the time; energy, and cost to produce tailored technologies that effectively address farmers’ problems. Thus, this research was conducted to determine nutritional quality, farmer preferences, and the relationship between local feed valuation and laboratory outcomes of indigenous legume fodder trees and shrubs (ILFTS) in a sub-humid environment.

## Materials and methods

### Description of the study area

**Figure 1:**
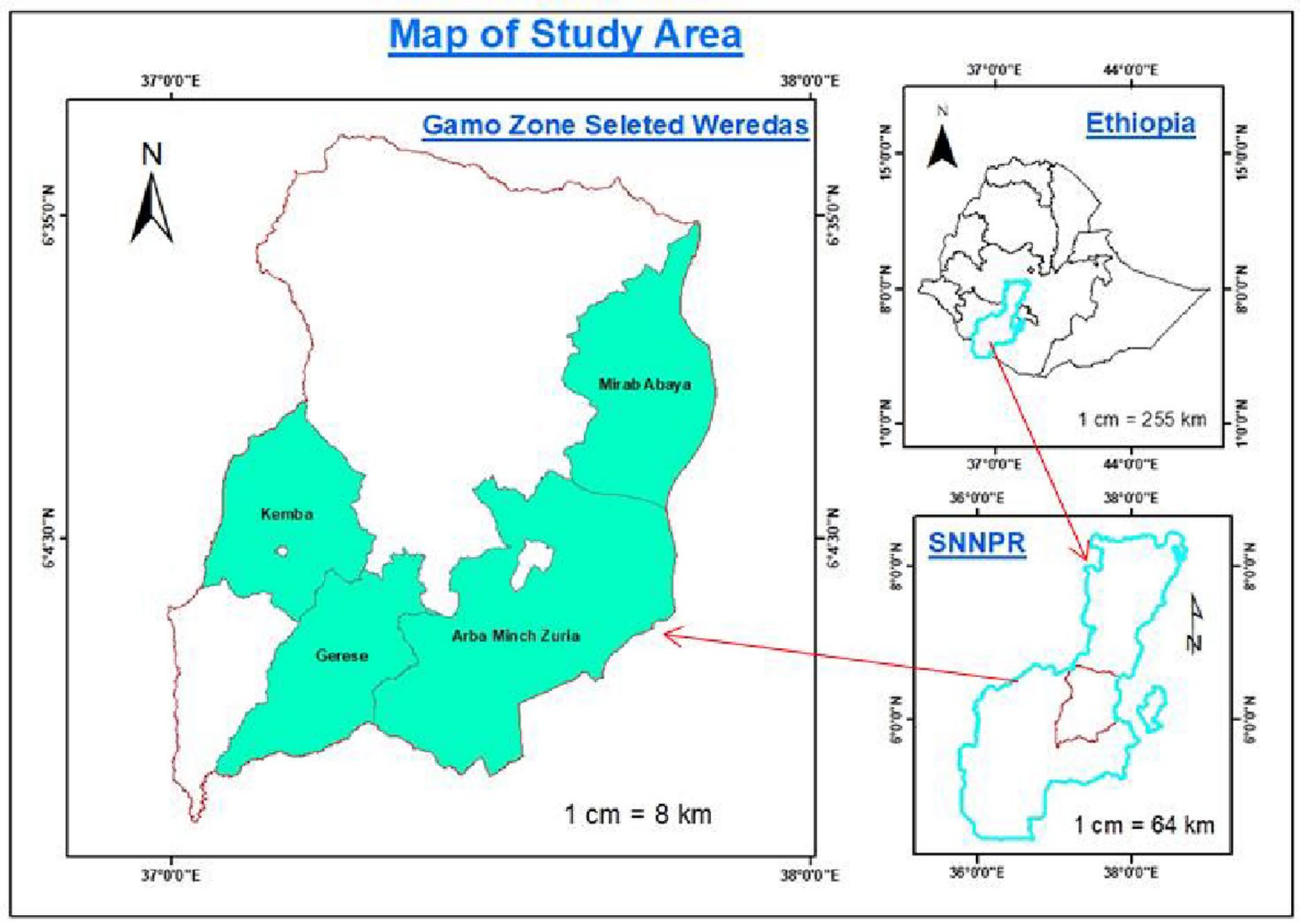
The map of the study districts in Gamo landscape.

The research was conducted in Gamo zone of southern Ethiopia, which is one of the sub-humid regions of the country. The zone lies between 5′57° – 6′71° North, latitude, and 36°37′–37°98′ East, longitude. The elevation in the Gamo zone ranged between 501 and 4,207 m above sea level, which is the reason for a varied climate and agroecosystems that produced wide biodiversity. Gamo zone is characterized by bimodal rainfall with the mean annual rainfall ranging from 801 to 2,000 mm and the annual mean temperature range from 10.1 to 27.5°C. The terrain has an undulating feature that favors the existence of different agro-climatic zones in close proximity ranging from dry lowlands to wet highlands. Mixed crop-livestock production is a prominent farming system where fruit and vegetable are the dominant crops in the lowland unlike in the highland where *Ensete ventricosum* predominates (16). Agroforestry is the common practice where trees are an integral component of the farming system that complements the function of land uses and enhances productivity (2) (see Figure 1).

### Farmers’ preference scoring and sample collection of ILFTS

Following a purposive sampling procedure, 60 experienced and acquainted farmers (20 in each agro-ecological zone) that were participating in the management and utilization of ILFTS were chosen for preference scoring. Focus group discussions (FGD) with 10 farmers were held in each agroecological zone to explore the desired tree characteristics and perceived benefits of ILFTS, which were the foundations to set the benchmarks for preference scoring. As a result, feed value, growth rate, biomass yield, compatibility, and multifunctionality were established as benchmarks for preference scoring of ILFTS. The nutritional preference score was determined based on criteria such as palatability, improvement of body condition, growth, and milk production, improvement of straw diet intake, and improvement in animal health, while the preference score for growth and regrowth potential was determined by criteria such as growth rate after establishment and re-growth potential after frequent cutting or looping. Farmer’s preference for compatibility was primarily based on the absence of crop competition for available soil nutrients and moisture, which improves soil fertility and improves the growth of annual and perennial crops below the canopy. Timber, poles, and other local constructions, as well as fuel wood, fences, medicinal values, shade trees, honey sources, soil stabilization, and farm implements, were incorporated to create multifunctionality indices. The rating of the ILFTS species was done using a preference score sheet. In each agro-ecological zone, preference scoring was carried out for ILFTS species on a point scale ranging from 1 (not preferred) to 4 (highly preferred) (17). Respondent farmers completed the ranking exercise on an individual basis. In each agro-ecological zone, samples of ILFTS, leaves, fruits, and pods were collected and were chemically analyzed. Five to ten individual plants per species were sampled and pooled to obtain a representative sample for each species in each agroecological zone. The samples were air-dried before being taken to the lab for testing.

### Chemical analyses of indigenous legume fodder trees and shrubs

The leaf (all ILFTS), pod (*Acacia tortilis*), and fruits (*Acacia albida*) samples were collected from the lowland [1,000–1,500 m above sea level (masl)], midland (1,500–2,300 masl) and highland (2,400–3,000 masl) in May and June. The dry matter (DM) content was determined by oven drying the feed samples at 55°C for 72 h for the constant weight (18). Oven-dried feed samples were ground using a Wiley mill to pass through a 1 mm sieve for chemical analyses. Contents of DM, total ash, and crude protein (CP) were analyzed following the standard methods of AOAC (18). The method of Van Soest et al. (19) was used to determine neutral detergent fiber (NDF). Acid detergent fiber (ADF) was determined following the method (20). Accordingly, the NDF and ADF analyses were followed sequentially. The residual ash was included in the NDF and ADF values. By solubilizing cellulose with 72% H_2_SO_4_, lignin (ADL) was determined (20). The difference between the percentages of NDF and ADF was used to calculate the hemicellulose (% HC). Total condensed tannins (CT) were determined using a butanol-HCl reagent and 2 percent ferric ammonium sulfate in a 2 N HCl catalyst (21). All chemical analyses were carried out in duplicate.

### *In vitro* dry matter digestibility potential of ILFTS

The two-stage technique was implemented to determine the *in vitro* dry matter digestibility (IVDMD) of leaf fruit and pod samples (22) as modified by Van Soest and Robertson (20). The rumen liquor was collected from three rumen cannulated steers before the morning feeding that was fed natural pasture hay (5%-6%CP) *ad libitum* supplemented with about 2 kg of concentrate (69% wheat bran, 30% noug seed cake and 1% salt) mixture per steer/day. The liquor from three steers was mixed on a volume basis and filtered through cheesecloth. The incubation inoculum was prepared by diluting the rumen liquor with a buffer solution (NaHCO_3_ + Na_2_HPO_4_ + KCl + NaCl + MgSO_4_. 7H_2_O + CaCl_2_.2H_2_O) (1:2 v/v) in a 1:4 (v/v) ratio (22). The mixed inoculum was stirred in a water bath at 39°C with purging CO_2_ until its use (10–15 min later). About 0.5 g (1 mm ground) of each sample was placed into 50 mL sterile tubes, and 20 mL of the incubation inoculum was added. The tube was stoppered with a Bunsen valve and incubated for 48 h at 39°C. Tubes were gently swirled by hand every 8 h. Each sample was incubated in three replicates. At the end of 48 h of the incubation period, the tube contents were acidified using 6 M HCL to reach a final pH of 1.3–1.5. After the foam subsided, the pepsin powder was added to the final concentration of 0.2% (w/v). Then, the sample was reincubated for 48 h again. The undigested portion of the sample (residue) was transferred into the crucible, and the liquid was filtered out via a sacking machine. The pellets were dried in a forced-air oven at 105°C for 24 h to determine the residual DM weight. Three blanks were included in each run. Then, to determine the ash content, the residues were kept at 550°C for 8 h to estimate ash. The *in vitro* DM and OM digestibility was determined as the DM and OM which disappeared from the initial weight added into the tube using the following equation.


$$\mathrm{IVDMD}\;\left(\%\right)=\left[\frac{(\mathrm{DM}\;\mathrm{sample}-\left(\mathrm{DM}\;\mathrm{of Residue}-\mathrm{Blank}\right)}{\mathrm{DM}\;\mathrm{of sample}\;}\right]\times 100$$

$$\mathrm{IVOMD}\;\left(\%\right)=\left[\frac{(\mathrm{OM}\;\mathrm{sample}-\left(\mathrm{OM}\;\mathrm{of Residue}-\mathrm{Blank}\right)}{\mathrm{OM}\;\mathrm{of sample}\;}\right]\times 100$$

Digestible organic matter in total dry matter (DOMD) was calculated as 0.95 IVDMD (%) − 2 (23). Metabolizable energy (ME) was estimated from DOMD (24) using the equation indicated below:


ME(MJ/kgDM)=0.016DOMD(g/KgDM)

where: DOMD = Digestible Organic Matter in the Dry matter.

### Statistical analysis

Data on the farmers’ assessment of ILFTS, and feed value preference score in agroecosystems, and the chemical composition including total CT, IVDMD, and ME values among trees shrubs, and fruits/pods in the lowland were subjected to the analysis of variance (ANOVA). The farmers’ preference score between trees and shrubs was analyzed using an independent t-test. Tukey HSD tests were used for mean separation. Mean differences were declared at *p* < 0.05. The analyses were performed using SPSS statistical software version 21 following the model indicated below:


$$Y_{ij}=\mu +c_{i}+dj+\epsilon_{ij}$$

where,

$Y_{ij}$ : Response variable; μ: overall mean effect;$\;c_{i}$: the effect of plant species; *dj*, the *j*th effect of agroecology and $\epsilon_{ij}$ is the random error.

Pearson correlation coefficient was done to analyze the relationships that exist, if any, between farmers’ feed value preference scores of ILFTS with the relative evaluation derived from laboratory-based indicators of feed quality.

## Results

### Socioeconomic characteristics’ of farmers

Socioeconomic characteristics’ of the respondents’ exhibited a wide variation among agroecosystems (Table 1). The sex and marital status of the respondents indicated that the majority were male (91.7%) of which 95% were married and the remaining were widowed. There was a variation in age category, with the majority of the respondents aged 41–50 years, followed by 31–40 years, with only 1.67% of the respondents below 30 years of age. The purposive sampling procedure intended to select the most knowledgeable respondents might favor the older and male groups. The educational level of the respondents varied widely, with the majority attending grades 5–8 (28.3%) followed by basic education (25%), where those attending above grade 12 were the least (3.33%). The limited education facilities in the area, which is a common occurrence in most rural parts of the country, might explain the low educational level of the respondents in the study. The land holdings of the majority of the respondents were 0.51–1 ha (36.67%) and 0.26–0.5 ha (30%), respectively. However, 10% of the respondents had less than 0.25 ha.

**Table 1:** The socioeconomic characteristics’ of the respondents’ (*N* = 60).

Parameter	Category	Agroecological zone			Total
		Lowland	Midland	Highland	
Sex of the respondents’	Male	18	17	20	55 (91.7%)
	Female	2	3	0	5 (8.3%)
Age category of the respondents’	21–30 years	1	0	0	1 (1.67%)
	31–40 years	6	7	6	19 (31.67%)
	41–50 years	7	8	10	25 (41.57%)
	Above 51 years	6	5	4	15 (25%)
Marital status of the respondents’	Married	19	18	20	57 (95%)
	Widowed	1	2	0	3 (5%)
Educational level of the respondents’	Illiterate	1	4	8	13 (21.67%)
	Basic education	5	8	2	15 (25%)
	Grade 1–4	3	2	3	8 (13.33%)
	Grade 5–8	7	6	4	17 (28.33%)
	Grade 9–12	3	0	2	5 (8.3%)
	Above 12	1	0	1	2 (3.33%)
Position of the respondent in the community	Locality admin	4	1	1	6 (10%)
	Spiritual leader	1	2	2	5 (8.3%)
	Elder	6	0	3	9 (15%)
	Ordinary farmer	9	17	14	40 (66.7%)
Land holdings	<0.25 ha	0	3	3	6 (10%)
	0.26–0.5 ha	4	9	5	18 (30%)
	0.51–1 ha	9	6	7	22 (36.67%)
	>1 ha	7	2	5	14 (23.3%)
Total		20	20	20	60 (100%)

### Farmers’ preference of ILFTS

The study indicated that farmers used multiple criteria to evaluate the ILFTS and the farmers’ feed value preference score exhibited significant differences between species in all agroecosystems and between shrubs and trees in the lowlands (Tables 2, 3). Among the trees *Acacia seyal* and *Acacia albida* (*A.albida*) excelled in feed value preference score however *Acacia nilotica* (*A.nilotica*) scored the least in the lowland. *Acacia brevispica* (*A.brevispica*) and *Acacia mellifera* (*A.mellifera*) excelled among the shrubs in feed value preference score in the lowland whereas *Dichrostachys cinerea* scored the least. In both midland and highland agroecosystems, *Albizia schimperiana* (*A.schimperiana*) and *Erythrina brucei* (*E.brucei*) showed significantly high feed value preference scores.

**Table 2:** Farmer preference score of ILFTS with species and evaluation parameters (*N* = 60).

Species/ agro-ecology	Feed value	Growth rate	Biomass yield	Compatibility	Multifunctionality	Overall mean
Lowland trees						
*Acacia tortilis*	3.53^ab^	2.1^c^	2.73^c^	2.60^e^	3.23^abc^	2.84^bc^
*Acacia seyal*	3.63^a^	2.00^c^	2.78^c^	2.53^e^	3.17^bc^	2.82^bc^
*Acacia albida*	3.57^a^	2.00^c^	2.7^c^	3.92^a^	3.28^ab^	3.09^a^
*Tamarindus indica*	3.07^d^	2.48^b^	3.55^a^	3.17^bc^	3.22^abc^	3.1^a^
*Aeschynomene elaphroxylon*	3.25^cd^	2.9^a^	2.9^c^	2.97^cd^	1.93^e^	2.79^c^
*Acacia polyacantha*	3.22^cd^	2.9^a^	2.95^c^	3.25^b^	3.08^c^	3.08^a^
*Acacia senegal*	3.68^a^	2.65^b^	2.88^c^	2.87^d^	3.29^ab^	3.07^a^
*Acacia nilotica*	2.6^e^	2.63^b^	2.78^c^	2.97^cd^	3.4^a^	2.87^bc^
*Acacia sieberiana*	3.32^bc^	2.05^c^	3.25^b^	3.23^b^	2.59^d^	2.89^b^
Mean	3.32	2.41	2.94	3.06	3.02	2.95
SD	0.39	0.4	0.38	0.44	0.48	0.15
Level of sign.	***	***	***	***	***	***
Shrubs						
*Acacia hockii*	2.98^b^	2.28	2.7^b^	2.75^b^	3.19^a^	2.78^bc^
*Dichrostachys cinerea*	2.72^c^	2.23	2.5^c^	3.05^a^	3.09^b^	2.72^c^
*Acacia mellifera*	3.68^a^	2.35	2.95^a^	3.23^a^	3.25^a^	3.1^a^
*Acacia brevispica*	3.61^a^	2.38	2.5^c^	3.05^a^	2.68^c^	2.84^b^
Mean ± SD	3.3 ± 0.47	2.3 ± 0.3	2.7 ± 0.3	3.02 ± 0.3	3.05 ± 0.2	2.86 ± 0.18
Significance level	***	NS	***	***	***	***
Midland trees						
*Acacia lahai*	2.98^d^	2.8^bc^	2.7^d^	2.95^b^	3.38^a^	2.97^d^
*Acacia abyssinica*	3.70^ab^	2.8^bc^	3.00^c^	3.05^b^	3.38^a^	3.2^ab^
*Piliostigma thonningii*	3.52^bc^	2.73^c^	3.25^b^	3.00^b^	3.40^a^	3.18^ab^
*Millettia ferruginea (M)*	2.88^d^	2.83^bc^	3.23^bc^	3.08^b^	3.50^a^	3.1^c^
*Albizia schimperiana (M)*	3.75^a^	3.00^b^	3.15^bc^	3.74^a^	3.41^a^	3.4^a^
*Erythrina brucei (M)*	3.71^a^	3.78^a^	3.63^a^	3.12^b^	2.69^b^	3.38^a^
*Erythrina abyssinica*	3.34^c^	3.63^a^	3.50^a^	3.10^b^	2.68^b^	3.23^b^
Mean ± SD	3.41 ± 0.39	3.08 ± 0.48	3.21 ± 0.37	3.14 ± 0.4	3.21 ± 0.34	3.21 ± 0.17
Significance level	***	***	***	***	***	***
Highland trees						
*Millettia ferruginea (M)*	3.55^b^	3.33^b^	3.0^b^	3.19	3.54^a^	3.44
*Erythrina brucei (H)*	3.81^a^	3.95^a^	3.5^a^	3.28	2.68^c^	3.44
*Albizia schimperiana (H)*	3.83^a^	3.88^a^	3.4^ab^	3.33	2.79^b^	3.45
Mean ± SD	3.73 ± 0.24	3.72 ± 0.38	3.5 ± 0.27	3.27 ± 0.2	3.0 ± 0.37	3.44 ± 0.1
Significance level	***	***	***	NS	***	NS

^a,b,c,d^The same subgroup in a column bearing different superscripts differ significantly; ***Significant at 0.001 level; NS, not significance; M, midland; H, highland.

**Table 3:** Farmers’ preference score between trees and shrubs of ILFTS in the lowland.

	Tree	Shrub	Mean	SD	Sign. level
Feed value	3.32	3.25	3.29	0.42	NS
Growth rate	2.41^a^	2.31^b^	2.38	0.38	*
Biomass yield	2.94^a^	2.66^b^	2.86	0.37	***
Compatibility	3.06	3.02	3.04	0.4	NS
Multifunctionality	3.02	3.05	3.03	0.42	NS
Overall mean	2.95^a^	2.86^b^	2.92	0.17	***

^a,b,c,d^The same raw bearing different superscripts differ significantly; NS, not significant, *Significant at 0.05 level; ***Significant at 0.001 level.

Likewise, among the parameters of tree characteristics, the farmers’ preference score for growth rate among the shrubs in the lowland, and compatibility and multifunctionality in the highland exhibited non-significant differences (*p* < 0.05) with species. The study, however, found a significant difference between shrubs and trees in the lowland (Table 3). Among the trees *Aeschynomene elaphroxylon* (*A.elaphroxylon*) and *Acacia polyacantha* (*A.polyacantha*) in the lowlands, *E.brucei* and *Erythrina abyssinica* (*E.abyssinica*) in the midland, and *E.brucei* and *A.schimperiana* in the highland excelled in growth rate.

Similarly, *Tamarindus indica* (*T.indica*) among the trees and *A.mellifera* among the shrubs in the lowland, *E.brucei* and *E.abyssinica* in the midland, and *Millettia ferruginea* (*M.ferruginea*) in the highlands revealed the highest significant (*p* < 0.05) score for biomass yield, whereas *A.albida* (3.92) among the trees in the lowland and *A.schimperiana* (3.74) midland scored the highest significant compatibility score although no significant difference was observed among the highland trees and between trees and shrubs in the lowland. According to respondents, *A.albida* (lowland) and *A.schimperiana* (midland) improved soil fertility and stability as well as increased crop yield and were therefore preferred in the farmland. Of course, all ILFTS species are likely to enhance crop yield via improved soil fertility and stability due to their ability to fix atmospheric nitrogen (2, 25). Likewise, *A.nilotica* and *A. mellifera* among the trees and shrubs, respectively, in the lowland and all midland species except *Erythrina species* and *M. ferruginea* (highland) achieved significantly high scores (*p* < 0.05) for multifunctionality, as the study revealed. However, there is no significant difference between trees and shrubs in multifunctionality (Table 3). ILFTS have been used for multiple functions such as local construction, firewood, charcoal, tool handlers, local furniture, traditional medicine, bee forage, and fencing were among those mentioned by the respondents. Moreover, the overall mean score of the ILFTS revealed significant differences (*p* < 0.05) with species among trees and shrubs in the lowland and midland. Four ILFTS species, namely *A.albida*, *T.indica*, *A.polyacantha*, and *Acacia senegal* (*A.senegal*) among the trees and *A.mellifera* among the shrubs in the lowland, and two species namely *A.schimperiana* and *E.brucei* in the midland exhibited the highest score for overall mean, even though the highland species exhibited no significant difference. Trees excelled shrubs in growth rate, biomass yield, and overall mean in the lowland among farmers’ preference score parameters (Table 3).

### Nutritional value parameters

Wide variations in nutritive values were found among the ILFTS species; however, there was no significant variation among trees, shrubs and fruit/pods in the lowlands (Tables 4, 5). As an example, the DM content of the ILFTS species ranged from 838.3 to 948.4 g/kg with *A. brevispica* leaf exhibiting the highest content followed by *E.brucei* leaf (M) (midland) and *M. ferruginea* leaf (H) (highland) respectively; *Acacia hockii* leaf (*A.hockii*) showed the lowest. The difference in ash content among the ILFTS was more than fivefold ranging from 25.9 to 134.2 g/kg DM, where *P. thonningii* leaf and *A.sieberiana* leaf recorded the highest and lowest among the trees respectively, whereas *A. hockii* leaf and *D. cinerea* leaf had the highest and lowest values among the shrubs. A similar tendency was observed in the variation of CP content among the ILFTS which spanned from 81.8 to 314 g/kg DM where *E.brucei* leaf (M) (midland) exhibited the top, succeeded by the highland *M.ferruginea* leaf (H) and *E.brucei* leaf (H) in that order, although *A.albida* fruit revealed the least. *A. mellifera* leaf and *D. cinerea* leaf exhibited the highest and lowest value of CP among the shrubs.

**Table 4:** Chemical composition and IVDMD of the leaves, fruits, and pods of ILFTS (g/kg DM) by species and agroecosystems.

ILFTS	DM (g/kg)	Ash	CP	NDF	ADF	HC	ADL	IVDMD	ME (MJ/KgDM)	CT (mg/g DM)
Lowland trees										
*Acacia tortilis* lf	902	98.7	202.0	329.0	123.0	206.0	95.1	591.0	8.87	5.17
*Acacia seyal* lf	906	63.1	220.4	282.2	158.1	124.1	68.6	575.1	6.63	1.65
*Acacia albida* lf	919.2	40.7	202.8	272.4	130.5	141.9	82.4	520.6	7.81	7.09
*Tamarindus indica* lf	905.7	81.8	158.7	447.2	189.5	257.7	72.3	673.5	10.1	1.94
*Aeschynomene elaphroxylon* lf	895.7	58.9	170.4	414.2	146.6	267.6	57.8	699.3	10.5	2.2
*Acacia polyacantha* lf	913.7	88.5	195.8	334.6	133.0	201.6	108.2	490.5	7.35	4.48
*Acacia senegal* lf	924.5	55.5	259.7	271.7	111.7	160.0	55.4	683.8	10.3	2.21
*Acacia nilotica* lf	839.6	77.8	100.4	501.7	257.1	244.6	220.1	303.6	4.55	5.27
*Acacia sieberiana* lf	919.9	25.9	204.4	211.7	164.5	47.2	89.6	538.9	8.08	3.57
Mean	903	65.7	191	341	157	183	94.4	564	8.5	3.7
SD	2.6	23	44	95	44	72	50	123	1.8	1.9
Shrub										
*Acacia hockii* lf	838.3	75.2	131.3	441.3	246.4	194.9	204.7	394.1	5.9	6.03
*Dichrostachys cinerea* lf	877.2	44.8	101.1	446.5	188.4	258.1	155.8	470.4	7.1	6.78
*Acacia mellifera* lf	923.6	51.5	240.9	305.2	126.6	178.6	53.4	669.5	10.0	2.67
*Acacia brevispica* lf	948.4	67.2	271.6	406.5	161.3	245.2	88.8	601.3	9.02	3.87
Mean	897	59.7	186	400	181	219	125.7	534	8	4.8
SD	4.9	14	83	66	51	38	67.7	124.5	1.9	1.9
Fruit/pod										
*Acacia albida* fruit	924.7	110.8	81.8	551.9	224.9	327.0	93.3	656.7	9.85	7.08
*Acacia tortilis* pod	912.6	37.2	118.6	421.0	260.3	160.7	100.6	529.7	7.95	6.79
Mean	919	74	100	487	243	244	97	593	8.9	6.9
SD	0.9	52	26	93	25	118	5.2	89.8	1.3	0.2
Midland trees										
*Acacia lahai* lf	871.1	60.7	137.0	406.9	167.8	239.1	136.8	469.7	7.04	3.78
*Acacia abyssinica* lf	919.5	53.9	229.5	224.6	139.6	85.0	87.5	543.2	8.15	1.95
*Piliostigma thonningii* lf	888.8	134.2	169.9	618.1	259.3	358.8	85.9	740.5	11.1	2.54
*Millettia ferruginea* lf (M)	936.8	60.1	206.3	437.0	238.7	198.3	100.3	526.0	7.89	2.91
*Albizia schimperiana* lf (M)	877.7	50.1	152.9	520.7	210.8	309.9	115.7	540.0	8.1	2.36
*Erythrina brucei* lf (M)	942.4	106.5	314.0	439.0	208.1	230.9	70.0	609.9	9.15	1.45
*Erythrina abyssinica* lf	941.1	71.7	155.9	427.2	317.5	109.7	83.1	486.1	7.29	2.65
Mean	911	76.7	195	439	220.3	218.8	97	559.3	8.4	2.5
SD	31,2	31.6	61.6	119.6	58.9	98.9	22.7	91,0.8	1.4	0.7
Highland trees										
*Albizia schimperiana* lf (H)	939.3	40.9	247.9	300.6	152.6	148.0	90.5	580.2	8.7	1.7
*Erythrina brucei* lf (H)	939.9	82.8	276.3	453.0	225.4	227.6	80.1	601.0	9.01	1.91
*Millettia ferruginea* lf (H)	941.6	89.0	272.9	444.9	228.7	216.2	83.8	589.6	8.84	1.8
Mean	940.3	70.9	265.7	399.5	202.2	187.3	84.8	590.3	8.85	1.8
SD	11.9	26.2	15.5	85.7	43	43	5.27	10.4	0.16	0.1

DM, dry matter; CP, crude protein; NDF, neutral detergent fiber; ADF, acid detergent fiber; HC, hemicellulose; ADL, acid detergent lignin; IVDMD, in vitro dry matter digestibility; CT, condensed tannin; ME, metabolizable energy; lf, leaf; M, midland; H, highland.

**Table 5:** The nutritional quality among tree, shrub, and fruit/pod of ILFTS (g/kg DM) in the lowland.

	Tree	Shrub	Fruit/pod	Mean	SD	Sign. level
DM (g/kg)	903	897	919	903	31	NS
Ash	65.7	59.7	74	65.2	24	NS
CP	191	186	102	177	60	NS
NDF	341	400	486	376	97	NS
ADF	157	181	243	175	51	NS
HC	183	219	244	201	69	NS
ADL	94.4	126	97	103	51	NS
IVDMD	564	534	593	560	114	NS
ME (MJ/Kg DM)	8.5	8	8.9	8.4	1.7	NS
CT (mg/g DM)	3.73	4.84	6.94	4.5	2	NS

DM, dry matter; CP, crude protein; NDF, neutral detergent fiber; ADF, acid detergent fiber; HC, hemicellulose; ADL, acid detergent lignin; IVDMD, in-vitro dry matter digestibility; ME, metabolizable energy; CT, condensed tannin.

The HC and ADL components had the most variance, fluctuating from 47.2 to 327 and 53.4 to 220.1 g/kg of DM, respectively, tracked by ADF and NDF. The ILFTS NDF value ranged from 300.6 to 618.1 g/kg DM, with *P. thonningii* leaf revealing the most, accompanied by *A. albida* fruit and midland *A. schimperiana* leaf (M), but *A. lahai* leaf revealing the least. The ILFTS ADF value ranged from 111.7 to 317.5, with *E. abyssinica* leaf having the greatest value, preceded by *A. tortilis* pod and *Piliostigma thonningii* (*P. thonningii*) leaf, and *A. senegal* leaf having the lowest. *P. thonningii* leaf had the highest HC, followed by *A. schimperiana* leaf (M) (midland) and *A. elaphroxylon* leaf; however, *A. abyssinica* leaf (midland) had the least HC*. A. nilotica* leaf has the highest *ADL* value, followed by *A. hockii* leaf and *Acacia lahai* (*A. lahai*) leaf, respectively, with *A. mellifera* leaf having the lowest.

The variation of IVDMD and ME was more than twofold, ranging from 303.6 to 740.5 g/kg DM and 4.55 to 10.5 MJ/kg DM, respectively. In terms of decreasing order of IVDMD, the ranking for the leaves of five ILFTS species was *P.thonningii* > *A.elaphroxylon* > *A. senegal > T.indica > A.mellifera* leaf respectively, however, *A.nilotica* leaf showed the least. In terms of ME, *P.thonningii* displayed the highest rate followed by *A.elaphroxylon* leaf and *T.indica* leaf, respectively, while *A. nilotica* leaf displayed the lowest level. Likewise the variation in CT content of ILFTS species forages was more than fourfold which ranged from 1.45 mg/g DM to 7.09 mg/g DM with *Acacia* albida lf and *Acacia* albida pod exhibiting the highest content followed by *Acacia tortilis* pod. However, *Erythrina brucei* lf (midland) exhibited the least.

### The correlation among nutrients and farmers feed value scores

The nutritive parameters of the ILFTS, such as CP (*r* = 0.768), IVDMD (*r* = 0.6), IVOMD (*r* = 0.565), ME (*r* = 0.6), and DOMD (*r* = 0.6), had a positive significant correlation with the farmers feed value score, unlike the ADL (*r* = −0.702) and CT (*r* = −0.543) which exhibited negative significant correlation (Table 6). Fiber components such as NDF (*r* = −0.314), ADF (*r* = −0.332), and HC (*r* = −0.19) had a negative non-significant connection with the farmers’ feed value score. However, the largest positive and negative Pearson correlation coefficients with farmers’ feed value assessments were found in CP (*r* = 0.768) and ADL (*r* = − 0.702) respectively.

**Table 6:** Pearson correlation between the nutrients and indigenous knowledge of feed value of the ILFTS in Gamo landscape.

	Ash	CP	NDF	ADF	HC	ADL	IVDMD	IVOMD	DOMD	ME (MJ/kg)	CT	Feed value
DM	−0.015	0.685^***^	−0.296	−0.139	−0.306	−0.781^***^	0.509^**^	0.455*	0.509^**^	0.509^**^	−0.385	0.615^**^
Ash		0.019	0.612**	0.318	0.612^**^	−0.005	0.286	0.447^*^	0.286	0.285	−0.087	0.028
CP			−0.434	−0.388	−0.315	−0.594^**^	0.403^*^	0.364	0.403^*^	0.402^*^	−0.648^***^	0.768^***^
NDF				0.714^***^	0.858^***^	0.323	0.031	0.149	0.031	0.031	0.126	−0.314
ADF					0.252	0.372	−0.297	−0.212	−0.297	−0.297	0.095	−0.332
HC						0.174	0.261	0.361	0.261	0.261	0.105	−0.190
ADL							−0.838^***^	−0.774^***^	−0.838^***^	−0.838^***^	0.526^**^	−0.702^**^
IVDMD								0.984^***^	1.000^***^	1.000^***^	−0.445^*^	0.600^**^
IVOMD									0.984^***^	0.984^***^	−0.422^*^	0.565^**^
DOMD										1.000^***^	−0.445^8^	0.600^**^
ME											−0.444^*^	0.600^**^
CT												−0.543^**^

DM, dry matter; CP, crude protein; NDF, neutral detergent fiber; ADF, acid detergent fiber; HC, hemicellulose; ADL, acid detergent lignin; IVDMD, in-vitro dry matter digestibility; IVOMD, in-vitro organic matter digestibility; DOMD, digestible organic matter in dry matter; ME, metabolizable energy; CT, condensed tannin; *Significant at 0.05 level; **Significant at 0.01 level; ***Significant at 0.001 level; *r*, Pearson correlation coefficient.

The CP of the ILFTS had a substantial positive connection with DM (*r* = 0.685), OM (*r* = 0.507), IVDMD (*r* = 0.403), DOMD (*r* = 0.403), and ME (*r* = 0.402), but a significant negative correlation with NDF (*r* = −0.434). The IVDMD had a substantial positive connection with DM (*r* = 0.509) and CP (*r* = 0.403), whereas the ADL had a significant negative correlation (*r* = −0.838). The IVOMD showed a positive significant link with DM (*r* = 0.455) and ash, in contrast to HC (*r* = 0.361) and ADL (*r* = −0.838), which showed a negative significant correlation. The ME showed a positive significant association with DM (*r* = 0.509), IVDMD (*r* = 1.0), IVOMD (*r* = 0.984), and DOMD (*r* = 1.0), but a negative significant link with ADL (*r* = −0.702).

The ADL had negative significant correlation with DM (*r* = −0.781), OM (*r* = −0.589), IVDMD (*r* = −0.838), IVOMD (*r* = −0.774), and DOMD (*r* = −0.984); however, it revealed positive non-significant correlation with the fiber components such as NDF (*r* = 0.323), ADF (*r* = 0.372) and HC (0.174).

The CT had a negative non-significant correlation with DM (*r* = −0.385), Ash (*r* = −0.087), and OM (*r* = −0.236) unlike the CP (*r* = −0.648) which revealed a negative significant correlation. CT exhibited negative significant correlation with IVDMD (*r* = −0.445), IVOMD (*r* = −0.422), DOMD (*r* = −0.445), and ME (*r* = −0.444) unlike the ADL (*r* = 0.526) which displayed positive significant correlation, though it showed positive non-significant correlation with NDF (*r* = 0.126), ADF (*r* = 0.095) and HC (*r* = 0.105).

## Discussions

### Farmers’ preference of ILFTS

The study indicated that farmers used multiple criteria such as feed value, multifunctionality, growth rate, biomass yield, and compatibility to evaluate the ILFTS which marked the farmers’ preference measures for the ILFTS as multifaceted (Table 2). This was due to the multiple functions of ILFTS as substantiated by several studies in the tropics and subtropics (2, 26). In agreement with the current study, various studies in Ethiopia have unveiled multiple criteria employed by farmers to evaluate fodder trees (4, 27); however, some emphasized certain criteria. For instance, availability and feed value were the major criteria to evaluate fodder trees in northwestern Ethiopia (4) and southern Ethiopia (28) due to critical feed shortages during the dry season. Some of the evaluation criteria used for the farmers’ preferential treatment of the ILFTS in the study are similar to those employed in other studies conducted in Ethiopia (4, 12), possibly because of the similarities in sociocultural practices.

The preference score was the farmer’s relative appraisal of one species over another on a given parameter. Distinct species of ILFTS inhabited the lowlands and highlands, even though the midland featured the common species of both agroecological zones, according to the study. The ILFTS species analyzed in the lowland, midland, and highland of the study were 13, 7, and 3, respectively, and hence the higher the species diversity across the agroecological zones, the higher the variation in the perception which in turn affects the preference rating and vice versa. Various research conducted in Ethiopia, in concurrence with the current study, showed that farmers’ choices for fodder trees varied significantly with species and evaluation criteria in agroecosystems (4, 12).

The superior feed value preference score of the respondents for the ILFTS in the lowland (*A. tortilis*, *A.seyal*, *A.albida*, *A.senegal*, *A.mellifera*, and *A.brevispica*), midland and highland (*E.brucei* and *A.schimperiana*) were probably due to the high perceived benefit of feeding them to ruminants, which enhances the quantity and quality of the diet as the study unveiled. Fodder trees are rich in CP, energy, and minerals (29, 30), adding to ruminants fed low-quality fiber-based diets enhanced their performance (8, 31). The higher performance of ruminants fed fodder trees and shrubs could potentially be attributed to reduced gastrointestinal distress and effective protein and energy use, because smaller quantities of secondary metabolites, particularly condensed tannins, are susceptible to such activities (reducing methane emission and enhancing bypass protein). The deleterious effect of CT on ruminant nutrition was substantiated by the negative significant correlation between CT and farmers’ feed value scores as revealed in the study. Various studies have discovered that fodder plants and shrubs contain anthelmintic (32, 33) and anti-methane emission constituents (34, 35). A high feed value score was observed for the *Acacia species* in Lay-Armachuho as well as the *Millettia* and *Acacia species* in Sidama (27) which was in contrast with the current study.

*A.polyacantha* (lowland), *E.brucei* and *E.abyssinica* (midland), and *E.brucei* and *A.schimperiana* (highland) excelled in growth rate due to their easily establishing nature and high growth potential that allowed reaching harvests (exploitation) early, make them valuable to farmers. Likewise, the ample and sustainable leaf yield of *T.indica* (lowland), *E.brucei* and *E.abyssinica* (midland), and *E.brucei* and *A.schimperiana* (highland) might justify their preeminence for biomass yield score. However, in a study conducted in southwestern Ethiopia (12), *E. abyssinica* had a low growth rate score, which is in contrary to the current study.

The ability of *A. albida* (lowland), *A.schimperiana* (midland), and highland species to enrich the soil and boost crop output growing underneath may explain why they are more compatible. Mekoya et al. (27) revealed the supremacy of the *Acacia species* for compatibility score in northern regions and southern semi-humid lands, which substantiate the current study, even though *A. abyssynica* in southwestern Ethiopia (12) exhibited significantly lower compatibility scores. In the current study, *A. nilotica* (lowland) and *M. ferruginea* (midland and highland) excelled for multifunctionality, likely due to their multiple functions such as poles or posts for construction, firewood, charcoal, farm implements, bee forage, and shade, in contrast to *Acacia* and *Millettia species* in northern dry midlands and Sidama (27), which scored slightly lower.

### Nutritional value parameters

The study found that there is a wide difference in nutritive value among the browse species, which is most likely due to the species’ inherent nature. Genomic and environmental factors influence the structure and composition of polysaccharides, such as lignin, cellulose, and hemicellulose resulting in differences in cell wall composition among forage species (36–38). The cell wall is comprised of 23%–90% of the plant tissue (37) hence its composition substantially affects the nutritive quality of forage. According to Grant et al. (39) and Ray et al. (40) regional and inter-annual climate variability causes fluctuations in forage nutritive values.

The ash values of most of the ILFTS species reported in the study are within the ranges reported for most native African browse species (10, 30). The ash values of *T.indica*, *A.nilotica*, and *A. tortilis* in the study were greater than the values observed in eastern Ethiopia (41). Likewise, the ash values reported for *A.polyacantha* in Tanzania are higher than the values found in the current study, yet *A.tortilis*, *A.nilotica*, and *Dichrostachys species* recorded lower amounts (42). The variation of the ash value of the ILFTS in the study compared to other studies is probably due to the soil fertility disparity, the season of harvest, and the harvesting stage of the fodder trees and shrubs (29, 36). Ash value is a potential indicator of the mineral content of forages however some minerals are volatile in nature hence further analysis is necessary to determine their actual value in the forages of ILFTS.

The CP values of most of the ILFTS species reported in the study are within the ranges found for most native African browse tree and shrub species (30, 43). While the CP value of *T.indica* leaf reported in the present study was similar to that observed in eastern Ethiopia; unlike, *A.tortilis* leaf and *A.nilotica* leaf which were higher and lower, respectively, than those in the current study (41). The study conducted in western Ethiopia reported 178 and 240 g/kg DM of CP for *A.abyssinica* leaf and *E.abyssyinica* leaf, respectively (12) which is lower and higher than the values found in the current study. The study revealed the minimum CP content of the ILFTS in the study was 81.8 g/kg DM, which was above the CP requirement [70 g/kg DM (44)] for normal rumen microbial function in ruminant livestock. The study substantiates that fodder trees and shrubs have the potential to complement the CP and mineral deficiency commonly observed in poor-quality pastures and crop residues, particularly during dry periods (45, 46).

The IVDMD values of most of the ILFTS reported in the study are within the range reported for native fodder trees and shrub species in Ethiopia and other tropical countries (10, 47). The IVDMD of *P.thonningii* leaf is the highest in the study, suggesting that it contains the maximum available nutrients among the ILFTS. The fiber of fodder trees and shrubs is more digestible than grass due to its less lignin content (5).

The NDF, ADF, and ADL values of some of the ILFTS species reported in the study are within the range reported for native fodder tree and shrub species in Ethiopia and other African countries (30, 41). For instance, the lignin value reported for *T.indica* leaf and *A.tortilis* leaf in the study was less than the value found in eastern Ethiopia (41) however the value for *A.nilotica* leaf was greater in the current study. The NDF and ADF values reported for *A.abyssinica* leaf and *E.abyssyinica* leaf in the study were lower and higher, respectively than the values found in western Ethiopia (12), unlike their ADL values. The variation in the fiber content of fodder trees and shrubs among studies is probably due to the harvesting season and stage of maturity of the browse trees (29)*. P.thonningii* leaf recorded the highest NDF value in the study implying likely to affect feed intake unlike *A.nilotica* leaf which showed relatively high NDF (520.7 g/kg DM) yet its ME value was the least due to its high ADL value. ADL is most likely to determine the nutritional value of plant fiber by interfering with the digestion of cell-wall polysaccharides by acting as a physical barrier. Li (38), Moore and Jung (48), and Yayneshet et al. (5) stated that lignin is the single most important cell wall constituent that impacts digestibility, which substantiates the present study. NDF, ADF, and ADL are plant cell wall fractions linked and packed together in tight configurations to resist degradation, and hence their nutritional value to animals varies substantially, depending on the composition, structure, and degradability (38).

### The correlation between nutrients and farmers’ feed value score

The farmers’ feed value preference score had a positive significant correlation with the nutritive value indicators DM (*r* = 0.615), OM (*r* = 0.458), CP (*r* = 0.768), IVDMD (*r* = 0.600), IVOMD (*r* = 0.565), DOMD (*r* = 0.600) and ME (*r* = 0.600), implying that farmers’ indigenous knowledge is relevant in judging the protein, energy, and digestibility values of browse species based on the perceived benefits associated with the animals’ performance measures. The greater the impact of feeding a certain browse species on animal performance, the higher the nutritional value, and hence the higher the grade. ADL (*r* = −0.702) and CT (*r* = −0.543) had a negative significant correlation with farmers’ feed preference score, indicating their impact on digestibility either through denying access or inhibiting microbial activity against cell wall components. Farmers used several indigenous criteria to judge the nutritional quality of available feed resources, according to Lumu et al. (49), including perceived effects on disease resistance, feed intake, growth/body condition, hair coat appearance, fecal output, and texture, and level of production, among others. Mekoya et al. (27) and Yisehak and Janssens (12) found a significant positive correlation of the farmers’ feed value score with the CP value of fodder trees and shrubs which partly agrees with the current study. The CP and IVDMD were positively correlated with the feed value score of the farmers in the highlands in the study conducted in northwestern Ethiopia (4), which agrees with the present study.

Because of its role in rumen microbial activity, CP showed a positive significant connection with IVDMD, DOMD, and ME. The ILFTS in the study had moderate to high CP values, which improved digestibility by increasing microbial activity. The high CP value of the ILFTS in the study suggests they could be used to supplement the N deficiency observed in ruminants feeding poor quality pastures and crop residues as a basal diet (26, 50).

Unlike ADF, which revealed a negative non-significant association with IVDMD, IVOMD, DOMD, and ME, ADL showed a strong negative significant link with IVDMD, IVOMD, DOMD, and ME. Both the IVDMD and IVOMD were depressed by the high ADL value of some of the indigenous browses in the research. The complex structure of plant cell walls, particularly the physical protection afforded by lignin, covalent connections between lignin and phenolic chemicals, and cell wall polysaccharides, impedes rumen digestion of fibrous plant components (51). Moore and Jung (48) discovered that lignin has a strong inhibitory influence on cell wall digestibility.

CT showed a negative significant connection with IVDMD, IVOMD, and DOMD, showing that it has a digestive inhibitory impact via suppressing microbial activity. Several investigations have revealed that CT is a secondary metabolite that binds the available CP in the rumen (9, 52) and hence lowers rumen microbial activity, affecting DM degradability. The CT and ADL have a positive connection, implying that they have a complimentary biosynthesis where the former is a secondary metabolite and the latter is a structural component.

## Conclusion

In the study, the ILFTS had a CP value over 81 g/kg DM confirming that it can be used to supplement ruminant diets that are deficient in N. In spite of this, there is a wide variation in nutritive quality among indigenous browse trees, probably because of the differences between species and agroecological zone which impact farmers’ preferences score. The farmer’s evaluation of ILFTS species was multidimensional, which encompasses the perceived benefits associated with animal performance measures and desired characteristics of a tree as they have been used for multiple purposes. Yet, the preference score for all evaluation parameters varied significantly with species except for growth rate in the lowland and compatibility and overall mean in the highlands. However, significant differences were observed in growth rate, biomass yield, and overall mean between shrubs and trees in the lowlands. The nutrients in ILFTS exhibited various correlations among themselves and with feed value preference scores depending on their distinct nature and biochemical function. Additionally, CP, CT, and ADL values of the ILFTS had significant correlations with IVDMD, thereby affecting the energy and protein supply of the ILFTS forage, as well as its feed value preference score as demonstrated by the animals’ performance measures. Thus, farmers’ indigenous knowledge of feed value may be relevant to for evaluating the nutritional quality of ILFTS forage by envisaging its nutrient content and interaction with other nutrients and may be used to complement scientific indicators.

## Data availability statement

The datasets presented in this study can be found in online repositories. The names of the repository/repositories and accession number(s) can be found at: https://doi.org/10.5281/zenodo.6585390.

## Author contributions

GA conceptualized the idea of the study, formulated the methodology, curated data, specimens of plant sample collection, performed formal analysis and investigation, wrote the original draft, and administrated the project. YK and DA reviewed and edited the manuscript. All authors contributed to the article and approved the submitted version.

## Conflict of interest

The authors declare that the research was conducted in the absence of any commercial or financial relationships that could be construed as a potential conflict of interest.

## Publisher’s note

All claims expressed in this article are solely those of the authors and do not necessarily represent those of their affiliated organizations, or those of the publisher, the editors and the reviewers. Any product that may be evaluated in this article, or claim that may be made by its manufacturer, is not guaranteed or endorsed by the publisher.
